# MARKERS OF PHYSICAL HEALTH AND HEALTH BEHAVIORS CONTRIBUTE TO LATER COGNITIVE FUNCTIONING AMONG ADULTS AGED 55–65

**DOI:** 10.1093/geroni/igae098.2013

**Published:** 2024-12-31

**Authors:** Ben Katz, Laura Sands, Maham Khan

**Affiliations:** Virginia Tech, Blacksburg, Virginia, United States; Virginia Tech, Blacksburg, Virginia, United States; Virginia Tech, Blacksburg, Virginia, United States

## Abstract

Life’s Essential 8 (LE8) represents a summary score consisting of a set of physical health (BMI, blood pressure, cholesterol, A1C) and health behaviors (sleep, diet, smoking status, physical exercise) that are closely associated with risk of cardiovascular disease. LE8 is designed to be simple for physicians to calculate, using regularly collected self-report and biomarkers, and easily interpretable for patients. While no equivalent score exists for Alzheimer’s disease and related dementias (ADRD), given close links between cardiovascular risk factors and cognitive outcomes, recent studies have identified links between LE8 and cognition and dementia. However, longitudinal work in this space is limited; additionally, many prior studies do not examine links from midlife to changes in later life cognitive functioning. The present study draws from 761 midlife adults aged 55-65 without cognitive impairment at baseline who were recruited to participate in the Health and Retirement Study. Data informing LE8-style indicators (including health behaviors and bloodspot biomarker data) were recorded in 2006. Performance on the Telephone Interview for Cognitive Status (TICS) assessment was measured 14 years later. Utilizing linear regression techniques that incorporated the HRS sample weights and age, gender, education, and baseline cognitive functioning, we found that both the aggregated health behaviors (p <.01) and aggregated physical health biomarker indicators (p <.001) accounted for unique variance in the TICS scores 14 years later. These findings have implications both for basic research to investigate the mechanisms connecting lifestyle and physical biomarkers to later cognition, but also for clinical practice and patient communication.

